# Carbohydrate Mouth Rinsing Improves Softball Launch Angle Consistency: A Double-Blind Crossover Study

**DOI:** 10.3390/nu17010167

**Published:** 2025-01-02

**Authors:** Tzu-Yuan Hsu, Meng-Hung Hsieh, Yi-Jie Shiu, Chih-Hui Chiu

**Affiliations:** 1 Management Doctoral Program, Daye University, Changhua 515, Taiwan; yuan691001@gmail.com; 2 Department of Physical Education, Tunghai University, Taichung 407, Taiwan; menghung@thu.edu.tw; 3 Physical Education and Sport Sciences, National Taiwan Normal University, Taipei 106, Taiwan; shiu880511@gmail.com; 4 Graduate Program in Department of Exercise Health Science, National Taiwan University of Sport, Taichung 404, Taiwan

**Keywords:** ergogenic aids, exit speed, launch angle

## Abstract

(1) Background: Carbohydrate mouth rinsing (CMR) stimulates the central nervous system and improves motor control. However, no studies have examined the effects of CMR on softball batting performance. The purpose of this study was to investigate the effect of CMR on softball batting performance. (2) Methods: Fifteen trained female collegiate softball players (age: 20.6 ± 0.9 years; height: 159.5 ± 5.2 cm; body weight: 58.1 ± 6.9 kg) completed two trials in a randomization crossover trail, in which they rinsed their mouths for 20 s with 25 mL of either 6.4% maltodextrin (CMR) or a placebo (PLA). After rinsing, the Posner cueing task and grip force, counter-movement jump (CMJ) and batting tests were performed in sequence. A tanner tee was utilized to hit five sets of five balls at a time, with a minimum 3 min rest between sets. The batting test recorded the average exit velocity, maximum exit velocity and launch angle consistency. The standardized standard deviation (SD) for launch angle represents the standardized variability. (3) Results: The consistency of the launch angle of the CMR trial was significantly greater (*p* = 0.025; Cohen’s d = 0.69) than that of the PLA trial. There were no significant differences in the Posner cueing task, grip strength, vertical jump, or exit velocity. (4) Conclusions: The findings of this study indicate that CMR enhances the launch angle consistency of all-out-effort batting, but does not influence the exit velocity of softball hitting.

## 1. Introduction

Many studies have found that pre-exercise carbohydrate supplementation is effective in maintaining high levels of glycogen in muscle and improving endurance performance in activities exceeding an hour in duration [1,2]. However, since Carter et al. (2004) revealed that the use of a carbohydrate mouth rinsing (CMR) still had a significant effect on improving athletic performance over a shorter period of time [3], it has been shown that CMR not only improves endurance performance over a short period of time, but also has a significant effect on high-intensity exercise [4], as well as improving resistance training quality [5] and the muscle strength of the lower limbs [6] and decreasing the fatigue index during intermittent exercise [7]. From these points of view, the CMR approach may contribute to the improvement of athletic performance.

CMR is defined as the use of a liquid containing about 6% carbohydrates, which is distributed around the mouth and then spat out. The mechanisms by which CMR improves athletic performance can be discussed in terms of both physiological and cognitive performance. It has been proposed that the mechanism of CMR involves the activation of the dorsolateral prefrontal cortex and the ventral striatum [8]. The cerebral cortex is integral to cognitive and attentional processing and motivation [9]. The aforementioned phenomena reveal that CMR not only benefits athletes in endurance sports but also enhances performance in explosive sports and improves body control during exercise. On the other hand, CMR has been found to activate the brain regions associated with exercise control [10]. This may resemble the effect of reducing central nervous system fatigue [11] and improving exercise performance. For strength performance, numerous studies have shown that CMR does not have an effect on single-repetition maximal muscle strength [12]; however, for repetitive contraction exercises, CMR has been found to be effective in improving peak power during repetitive Romanian deadlift exercises in the lower body [6]. Whether such a result would affect the hitting ability of fast-pitch softball players is one of the questions to be explored.

In the context of fast-pitch softball, enhancing both batting exit velocity and bat control indicates a crucial strategy for increasing both the scoring and winning rate of the game [13]. In each swing, it is necessary to combine speed and explosive power, which can be achieved through the use of lower extremity forces that are employed to transfer weight from the lower to the upper body. The rotation of the torso at a rapid pace also serves to increase the speed of the swing [14]. It has been suggested that increases in muscle strength and explosive power are viewed as important factors for improving the exit velocity of the ball for female softball players [15]. Although information on the effects of CMR on improving explosive exercise performance is still inconsistent, it is clear that CMR is effective in improving cognitive functioning [9], which may have an impact on bat control and batting accuracy. From these perspectives, CMR appears to be a promising approach for enhancing both exit velocity and batting control.

CMR has positive effects on athletic performance and activates areas of the brain related to motor control [10]. For softball batting, an increase in batting speed and stable control of the angle of elevation of the bat will improve batting performance and thus increase the team’s chances of winning. Nevertheless, there are no studies examining the effect of CMR on batting performance in softball. Therefore, the purpose of this study was to investigate the effect of CMR on the exit velocity and bat control of female softball players. The hypothesis of this study is that CMR can improve batting performance in trained softball players.

## 2. Materials and Methods

### 2.1. Experimental Design

**Figure 1:**
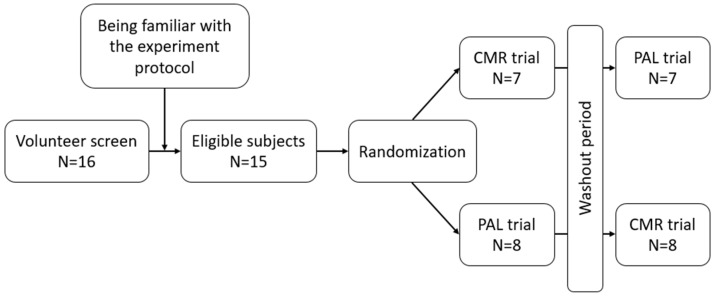
CONSORT diagram and study design.

This study was conducted by using a double-blind randomized cross-over experimental design, whereby all participants were required to complete two trials (Figure 1). The carbohydrate mouth rinsing trial (CMR) utilized a colorless and odorless water solution comprising 6.4% maltodextrin. Mineral water was used for the placebo trial (PLA). For each rinsing, 25 mL of the water solution was used, and the rinsing process was conducted for 20 s before the solution was spat back into the original cup. After the first rinse, participants were asked verbally if they could guess the trial that contained the carbohydrate. Of the 32 enquiries in the two trials, correct guesses were only made 15 times (46%), showing that the solution can successfully create a blind test effect. After completing the first experiment, washout periods were conducted with an interval of at least 7 days, and then, we completed the next experiment. This study lasted from 30 May 2022 to 30 August 2022. This was the out-of-season period for players. After the 5-day training period, the participants were given a 2-day break, and the experiment was carried out on the day after the break.

### 2.2. Participants

A total of 16 female collegiate fast-pitch softball players were recruited in this study. Following the withdrawal of one participant due to personal reasons, the final results of 15 participants (mean age: 20.6 ± 0.9 years; height: 159.5 ± 5.2 cm; mass: 58.1 ± 5.9 kg; mean training age: 6.8 ± 0.8 years) were analyzed. The inclusion criteria for this study were as follows: (a) more than 6 years of professional fast-pitch softball training; (b) more than 6 months of continuous training; (c) from at least the top four teams in the country. The exclusion criteria were as follows: (a) non-professional fast-pitch softball training; (b) a lack of regular training in the past 6 months; (c) the applicant had sustained a sports injury or was currently experiencing epilepsy, hypertension, hyperlipidemia, heart disease, arthritis, osteoporosis, or a brain injury, and had yet to undergo a period of recuperation exceeding three months. All participants signed consent forms after being informed of the procedures and the associated risks prior to the experiment. All tests were completed during non-menstrual periods. The study was approved by the institutional review board of China Medical University Hospital, Taiwan (CMUH110-REC2-253), and registered on ClinicalTrials.gov (Date: 16 October 2024; ID “NCT06659185”; https://register.clinicaltrials.gov). This study followed the principles of the Declaration of Helsinki and the recommendations proposed by the CONSORT Statement.

### 2.3. Sample Size Calculation

G*Power software was used (version 3.1.9.4, Universität Düsseldorf, Düsseldorf, Germany) [16] to determine the required sample size. The calculation was based on an alpha level of 0.05 and a correlation coefficient of 0.80, which were deemed to be the appropriate levels for the purposes of this study. A previous study demonstrated that CMR enhances lunge test accuracy in fencing, with an effect size of 0.40 [17]. The analysis revealed that a sample size of 15 would be sufficient for the purpose of detecting a difference between trials. Therefore, in this study, the sample size of 15 participants should be sufficient to elucidate the statistical discrepancies.

### 2.4. Protocol

The formal examination comprised grip strength assessments, counter-movement jump (CMJ) and batting tests. It was imperative that all participants underwent familiarization tests on at least two occasions prior to the formal test. Three days prior to the first formal trial, the participants were asked to photographically record the contents of all meals as well as the time of consumption. In the next formal experiment, the participants were asked to have exactly the same food at the same time. On the day of the experiment, after a standard lunch at 12:00, the participants arrived at the laboratory at 15:30 for the experiment.

The participants performed the first rinse followed by the Posner cueing task, which took approximately 8 min to complete. After a five-minute break, the participants were free to warm up dynamically. All warm-up movements and times were recorded and the same movements were used for the next warm-up. After warming up sufficiently, the participants performed the rinsing, which was followed by a maximum grip strength test with the dominant hand, where the participants performed a total of 3 sessions with a 1 min break between each session. Following the completion of the grip strength test, each of the participants was permitted a three-minute period of rest. Subsequently, they rinsed their mouths and proceeded to undertake the CMJ test on 3 occasions, with a one-minute interval between each attempt. In previous studies, a positive correlation has been identified between grip strength, jump height and the exit velocity of the ball in baseball players [18]. After completing these tests, participants underwent a 5 min dynamic batting warm-up. A tanner tee was utilized to hit five sets of five balls at a time, with a minimum 3 min rest between sets. The participants rinsed their mouths before each batting test.

### 2.5. Outcome Measure

The present study used the Posner cueing task as an indicator of cognitive functioning [19]. This test primarily assesses attention and was demonstrated to be an effective discriminator between high-performing and intermediate players in a previous study [20]. The participants were instructed to direct their gaze towards the central mark exhibited on a screen. Two boxes, one situated to the left of the center mark and another to the right, were clearly displayed. After a brief interval, a prompt was presented within the box, followed by the command “GO”. The participants were instructed to press the designated button (A/L) in accordance with the location of the command. This action resulted in the test displaying the command 100 times. The instances of congruent responses (on the same side as the instruction), the instances of incongruent responses (on the opposite side to the instruction) and the accuracy were all recorded [20].

In the grip strength test, the participants assumed a standing position with a standardized hand-held grip dynamometer (Smedlay’s Hand Grip Dynamometer TTM, Tokyo, Japan) and adjusted their grip to the second knuckle, with the arm at an approximately 10° angle to the trunk. Each participant was instructed to take the position of the grip at an angle of between 0 and 15 degrees to the trunk and to press the grip continuously for approximately three seconds with maximum strength, maintaining stability of the trunk throughout the procedure. The procedure was repeated three times, with a one-minute rest interval between each repetition, and the resulting data were averaged [21].

A countermovement jump test was conducted by utilizing a jumping mat (Fusion Sport, Coopers Plains, QLD, Australia) to evaluate the participants. The participants were instructed to maintain a static standing position on the jumping mat with their arms folded at the waist. The subjects were later given instructions by the instructor and then maintained a position with their arms akimbo, before rapidly squatting to approximately 90° knee flexion before performing a maximal vertical jump. The hip and knee joints were maintained in an extended position throughout the jump. Trials where the knee was flexed to an angle of less than 90 degrees or where the foot in contact with the ground were excluded from the analysis. Each participant completed three trials with a one-minute interval between each trial. The mean value was then calculated [22].

In the batting test, a tanner tee was used. The tanner tee was placed on the front edge of the center of the plate and the height of the tanner tee was adjusted to approximately the navel of the test participants. We set the batting target at a 10-degree angle, 10 m in front of the plate, and asked the batters to hit each ball to the target, with 5 swings per set, for a total of 5 sets. Hitting data were collected by using Rapsodo Hitting 2.0 (Rapsodo Bassball System, Rapsodo Inc., Fishers, IN, USA). This instrument has been employed in previous studies and has demonstrated sufficient reliability for the measurement of exit velocity and launch angle [19]. A batting test evaluates the exit speed of the softball along with the launch angle and calculates the standard deviation of launch angle, which is commonly used as an indicator of batting consistency [23].

### 2.6. Statistical Analysis

The data from this study were presented as the mean ± standard deviation and the analysis was undertaken by using the statistical software package SPSS 23.0. The normality of the data was evaluated using a Shapiro–Wilk test. The data were presented according to a normal distribution and a parametric methodology, and could thus be applied to the statistical analysis. Paired samples t-tests were employed to ascertain the statistical significance of the observed differences in the Posner cueing task, the hand grip strength, the countermovement jump, the average batting exit velocity, the maximum batting exit velocity and the SD of the launch angle. The magnitude of the observed effects was quantified using Cohen’s d, and a power analysis for each data set was conducted using G*Power 3.1.9.6 software [16]. The significance level was set at α < 0.05.

## 3. Results

### 3.1. The Launch Angle and Batting Accuracy

**Figure 2:**
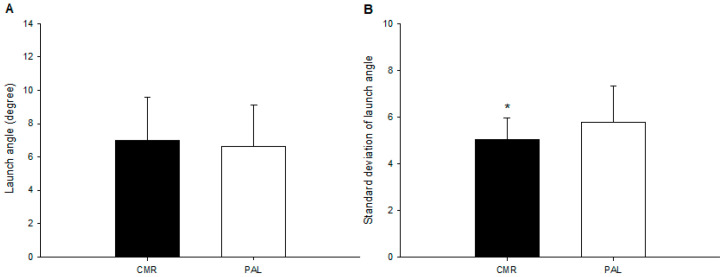
The launch angle and batting accuracy. The launch angle ((**A**); *n* = 15) and batting accuracy ((**B**); n = 15) of the CMR and PLA trials were compared. The values are the mean ± SD. CMR—carbohydrate mouth rinsing trial; PLA—placebo trial. * The CMR results were significantly better than those of the PLA.

There was no significant difference in the average launch angle (CMR: 7.0 ± 2.6 degree; PLA: 6.6 ± 2.5; *p* = 0.420; Figure 2A) between the two trials. Nonetheless, the consistency of the launch angle of the CMR trial was significantly greater (CMR: 5.0 ± 0.9; PLA: 5.8 ± 1.5; *p* = 0.025; Cohen’s d = 0.69; actual power = 0.81; Figure 2B) than that of the PLA trial.

### 3.2. The Posner Cueing Task

**Figure 3:**
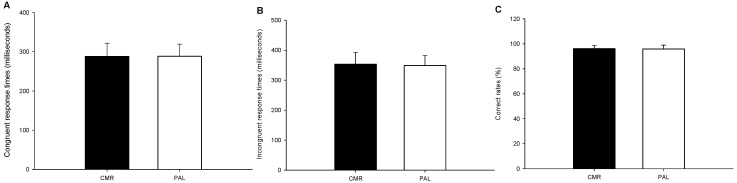
The results of the Posner cueing task. The response instances of a congruent response ((**A**); n = 15) and of an incongruent response ((**B**); n = 15), and the rate of correct responses ((**C**); n = 15) in the CMR and PLA trials were compared. The values are the mean ± SD. CMR—carbohydrate mouth rinsing trial; PLA—placebo trial.

There was no significant difference in the congruent response instances (CMR = 288.0 ± 33.59 ms; PLA = 288.6 ± 31.04 ms; *p* = 0.926; Figure 3A), incongruent response instances (CMR = 353.4 ± 39.3 ms; PLA = 348.8 ± 33.4 ms; *p* = 0.515; Figure 3B) or rates of correct answers (CMR = 96.07 ± 2.66%, PLA = 95.87 ± 3.10%, *p* = 0.810; Figure 3C) in the Posner cueing task.

### 3.3. Grip Strength and Countermovement Jump

**Figure 4:**
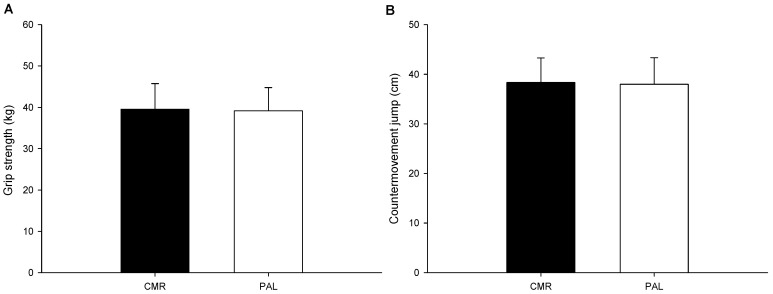
The grip strength and the jump height of the countermovement jump. The grip strength ((**A**); n = 15) and the jump height of the countermovement jump ((**B**); n = 15) in the CMR and PAL trials were compared. The values are the mean ± SD. CMR—carbohydrate mouth rinsing trial; PLA—placebo trial.

The results showed that there was no significant difference in grip strength (*p* = 0.601; Figure 4A) and the jump height of the countermovement jump (*p* = 0.777; Figure 4B) between the CMR and PLA trials.

### 3.4. The Exit Velocity

**Figure 5:**
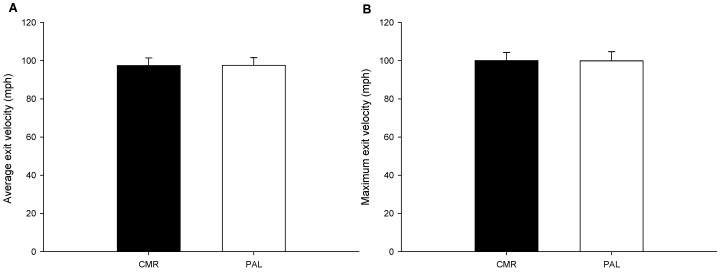
The exit velocity. The average exit velocity ((**A**); n = 15) and maximum exit velocity ((**B**); n = 15) in the CAF and PLA trials were compared. The values are the mean ± SD. CMR—carbohydrate mouth rinsing trial; PLA—placebo trial.

According to the results of the hit-off-tee, there were no statistically significant differences between the average (CMR: 97.4 ± 3.99 mph; PLA: 97.5 ± 4.01 mph; *p* = 0.754; Figure 5A) and maximum (CMR: 100.0 ± 4.0 mph; PLA: 99.9 ± 4.8 mph; *p* = 0.822; Figure 5B) exit velocity in the CMR and PLA trials.

## 4. Discussion

The practice of carbohydrate mouth rinsing prior to exercise has been linked to enhanced strength, power, and limb control. This study represents the first investigation into the potential impact of carbohydrate mouth rinsing on the batting performance of trained female fast-pitch softball players. Despite the lack of statistically significant differences in the Posner cueing task and batting performance in terms of exit velocity, this study still revealed that CMR led to a significant enhancement in the launch angle consistency of batting. It can be hypothesized that utilizing the CMR prior to hitting may enhance batting stability and control.

The use of CMR was found to be effective in activating movement control areas in the brain in a previous study. In this study, the standard deviation of the bat launch angle was calculated as an indicator of bat elevation consistency, which was used as a basis for bat control. The findings indicated that CMR was an effective method for enhancing the consistency of bat elevation angle, which may signify a considerable advancement in bat control during batting. In a previous study, Rowlatt et al. recruited fencing athletes as participants and discovered that CMR was an efficacious method for enhancing the precision of fencing athletes when executing an attack during fatigue conditions [17]. To the best of our knowledge, this is the only existing study that has found that CMR can be used in exercise for limb control. Moreover, the authors suggested that the lack of precision in past experiments may have contributed to the lack of significant differences in the effects of CMR on sport-specific performance [17]. Therefore, we included more precise measurements carried out Rapsodo Hitting 2.0 (Rapsodo Bassball System, Rapsodo Inc., Fishers, IN, USA) with a high-speed camera as well as a Doppler radar, and found that there was a significant difference in the consistency of the elevation angle of the ball. The findings of this study provide further evidence that CMR is an effective method for improving the launch angle consistency in batting. Yet, the exact mechanism remains unclear.

In the present study, the Posner cueing task, an attention test, was employed in order to investigate the mechanisms that are related to the accuracy of batting. However, there were no significant differences between the two trials in any of the indicators. In the case of softball batting, both visual attention and space variability are considered to be important factors. However, it should be noted that different cognitive tests may elicit different responses in different regions of the brain [8]. From these data, it seems that CMR does not have any effect on attention-related cognitive abilities. Therefore, this study, although it is unlikely to improve our understanding of the precise reasons for improving the launch angle consistency of batting, provides scientific evidence that CMR can be effective in increasing accuracy in batting.

The findings of this study indicate that CMR has no significant effects on either maximum exit velocity or average exit velocity during tanner tee hitting. Though it has been found that CMR appears to be effective in improving maximal muscle strength in the lower extremities, as well as explosive power [6], in previous studies, a recent systematic review with a meta-analysis indicated that CMR does not have a significant effect on explosive power in a single movement, nor does it improve athletes’ ability to perform repetitive movements at high intensities [12]. In this study, no significant differences were found in grip strength and jump height in a CMJ. Furthermore, only five balls were hit at a time during the batting test, and the participants were permitted sufficient rests during the batting process, so they showed no indications of fatigue. The data presented in this study provide new scientific evidence that it is not physiologically possible to significantly improve the exit velocity of the ball in the absence of fatigue while using the CMR method. Given that softball is also a sports competition that requires defense and sprinting, it is possible that fatigue may be an influencing factor in the second half of the game. Future studies may be conducted to induce fatigue and then intervene with CMR to ascertain whether there is a notable enhancement in exit velocity in the context of fatigue.

It is also pertinent to consider the fact that this study recruited trained female athletes as the participants. Previous studies have often employed male participants in order to circumvent the potential influence of factors such as the menstrual cycle on the results. In this study, all participants avoided performing the experiment during their menstrual cycle. This is because the menstrual cycle is an external factor that could have influenced the results of the study. In fact, according to a systematic review and meta-analysis study, it was found that there was no gender difference in the effect of CMR on exercise performance [24]. These results support the notion that the launch angle consistency of the ball improves after CMR, although there is no significant difference in the strength or power performance of the professionally trained female players.

The strength of this study is that it is the first study to recruit elite active female softball players to observe the effect of CMR on hitting ability, and it was found to be effective in improving accuracy in batting. Although the CMR method utilized in this study was consistent with those previously documented in the literature, including fasting time, mouth rinsing time, and the frequency of rinsing prior to the test [6], a limitation of this study is that it did not use methods such as functional magnetic resonance imaging (fMRI) to investigate the changes in the cerebral cortex after CMR. Conversely, in order to ensure greater consistency with the point of impact, the test was conducted by using tanner tee hitting rather than utilizing alternative techniques, including pitching machines. Future research may be able to conduct impact tests using pitching or pitching machines to further understand the effect of CMR on batting accuracy. In addition, a control trial in which the participants did not rinse was not included in this study, which may make it impossible to judge whether a learning effect was present. We concluded that a learning effect did not exist for the participants for two reasons: 1. the participants were professionally trained and were very familiar with the batting motion; 2. re-comparison of the experimental sequence showed a *p*-value of 0.64 for the consistency of the launch angle.

## 5. Conclusions

The findings of this study indicate that CMR is an effective method for enhancing the launch angle consistency of ball hitting, but it was observed that CMR was not as effective in improving the exit velocity when the players were not experiencing fatigue. Future studies could investigate potential of fatigue interventions, or consider alternative approaches, for instance, pitching or pitching machines, to gain an understanding of the ways in which CMR can be utilized in actual competitive situations.

## Data Availability

All relevant materials are presented in the present manuscript.
